# Masticatory Muscle Thickness and Activity Correlates to Eyeball Length, Intraocular Pressure, Retinal and Choroidal Thickness in Healthy Women versus Women with Myopia

**DOI:** 10.3390/jpm12040626

**Published:** 2022-04-13

**Authors:** Grzegorz Zieliński, Marcin Wójcicki, Maria Rapa, Anna Matysik-Woźniak, Michał Baszczowski, Michał Ginszt, Monika Litko-Rola, Jacek Szkutnik, Ingrid Różyło-Kalinowska, Robert Rejdak, Piotr Gawda

**Affiliations:** 1 Department of Sports Medicine, Medical University of Lublin, 20-093 Lublin, Poland; piotr.gawda@umlub.pl; 2 Independent Unit of Functional Masticatory Disorder, Medical University of Lublin, 20-093 Lublin, Poland; marcin.wojcicki@umlub.pl (M.W.); monikalitkorola@umlub.pl (M.L.-R.); jacek.szkutnik@umlub.pl (J.S.); 3 Students’ Scientific Association at the Department and Clinic of General and Pediatric Ophthalmology, Medical University of Lublin, 20-093 Lublin, Poland; maria.rapa00@gmail.com; 4 Department of General and Pediatric Ophthalmology, Medical University of Lublin, 20-093 Lublin, Poland; anna.wozniak@umlub.pl (A.M.-W.); robert.rejdak@umlub.pl (R.R.); 5 Interdisciplinary Scientific Group of Sports Medicine, Department of Sports Medicine, Medical University of Lublin, 20-093 Lublin, Poland; m.baszczowski@gmail.com; 6 Department of Rehabilitation and Physiotherapy, Medical University of Lublin, 20-093 Lublin, Poland; michal.ginszt@umlub.pl; 7 Department of Dental and Maxillofacial Radiodiagnostics with Digital Dentistry Lab, Medical University of Lublin, 20-093 Lublin, Poland; rozylo.kalinowska@umlub.pl

**Keywords:** eye, optometry, sEMG, eyeball length, retinal, choroidal, intraocular pressure, USG, masticatory muscles, myopia

## Abstract

This study aims to examine the correlations between masticatory and neck muscle thickness and activity versus eyeball length, retinal thickness, choroidal thickness, and intraocular pressure in healthy women versus women with myopia. The study group consisted of 21 women aged 24 years and a control group of 19 women (mean age 23 years). For bioelectrical activity analysis within the temporalis anterior, the superficial part of the masseter muscle, the middle part of the sternocleidomastoid muscle, and the anterior belly of the digastric muscle, an eight-channel BioEMG III electromyograph were used. An M-Turbo ultrasound machine was used to analyze masticatory and neck muscle thickness. The eyeball length was examined by IOL Master 500; choroidal and retinal thickness by Optovue Angiovue; and intraocular pressure by Tono-Pen XL. Refractive errors are related to differences in muscle thickness and electromyographic activity. Bioelectrical activity within the temporalis anterior seems to be associated with ocular length, retinal thickness, and choroidal thickness in women with myopia.

## 1. Introduction

The highest rate of increase in myopia is claimed to occur between the ages of 20 and 30. It is estimated that this would affect 72% of individuals and then decrease to 22% in patients over the age of 70. The peak of myopia occurs at the age of 24 [1]. It is predicted that by 2050, there will be 4.8 billion myopic patients [2]. The etiology of myopia is not fully understood and has a multifactorial character. Evidence suggests that not only genetic factors but also environmental factors, such as time spent outdoors, play a major role in the occurrence of myopia [3]. Short-sightedness involving blurring of objects viewed from a distance is related to refractive error, which results from a mismatch between different optical elements of the eye, one of which is the length of the eyeball [4]. The main form of myopia is axial-length myopia, in which the eyeball expands too much. The eyeball length increases during childhood and adolescence, and if this increases the eyeball axial length, it can exceed the focus of the eye leading to myopia [5]. For example, high myopia is defined as a refractive error ≤−6. 00 diopters (D) or an axial length ≥26. 5 mm [6].

Myopia is known to be associated with several ocular complications such as retinal detachment, glaucoma, cataract, optic nerve disc changes, and maculopathy [7]. These are not the only changes in the structure of the eye associated with myopia. Some studies suggest that intraocular pressure (IOP) is associated with the occurrence of refractive error [8,9]. Based on the Mendel randomization model, it was noted that on average, each 1 mm Hg increase in IOP predicts a decrease in the spherical equivalent of 0.05 to 0.09 Diopters (Dsph) [9]. This suggests that exposure to higher IOP may inadvertently increase the prevalence of myopia [10].

Additionally, there are also changes in the thickness of the retina and choroid in myopia. An overall reduction in retinal thickness has been observed in eyes with myopia compared to eyes without myopia [11,12,13]. Choroidal thickness in high myopia is inversely correlated with age and myopia refractive error and is an important predictor of visual acuity [14].

An increasing number of studies have recognized changes in the bioelectrical activity of masticatory and cervical spine muscles [15,16,17,18]. The connection between the eye and the masticatory muscles may have a neurological, biomechanical, or biochemical basis. Changes in electromyographic activity within masticatory muscles can have a primary or secondary character on the refractive error. However, the presented connection is not yet fully understood [17].

This study aims to examine the correlations between masticatory and neck muscle thickness and activity versus eyeball length, retinal thickness, choroidal thickness, and intraocular pressure in healthy women versus women with myopia. To the best of our knowledge, this is the first study to analyze the relationship between these systems.

## 2. Materials and Methods

### 2.1. Study Population

A total of 131 women in the age range of 20–30 years were enrolled in the study. The analysis in this study involved Caucasian women. It was decided to examine women due to the higher prevalence of myopia [19,20], faster progression of myopia in women [21], and higher prevalence of TMD [22,23] compared to men. The following exclusion criteria were used: the presence of TMD based on The Research Diagnostic Criteria for Temporomandibular Disorders (RDC/TMD), open bite, crossbite, Angle class II and III, lack of four spheres of support, oral inflammation, hyperopia, diseases of the optic nerve and ocular structures, neurological disorders of the head and neck, muscular disorders or diseases, neoplastic diseases (regardless of type or location), pain and trauma and previous surgical treatment in the head and neck within the last six months of the study, and pregnancy.

The examination for exclusion criteria was carried out by the authors: a dentist with a specialization in dental prosthetics (author J.S.), a medical doctor specialized in ophthalmology (author A.M.-W.), and a physiotherapist (author G.Z.).

Based on the above criteria, 40 women were included in the study. Refractive error based on ophthalmological examination (author A.M.-W.) was found in 21 subjects. The study group included women with a refractive error from −0.75 to −5.50 Dsph. The ophthalmological examination showed a refractive error in the right eye mean of −3.00 ± 0.50 Dsph and in the left eye mean of −3.00 ± 1.50 Dsph within the study group. The best-corrected visual acuity (BCVA) was tested using a Snellen chart, and it was 1.0 (20/20)). No refractive error was found in the control group (19 women). The uncorrected visual acuity was 1.0 (20/20). Other ophthalmic parameters are listed in Table 1. No other ocular abnormalities were found on a complete ophthalmological examination.

After the initial measurements and qualification to the study, the study group consisted of 21 women aged 24 years (±3 years) and 19 controls aged 23 years (±2 years). The groups were not statistically significantly different regarding age and number of participants (*p* = 0.25).

The study was conducted by the recommendations of the 1964 Declaration of Helsinki and its 2013 Seventh Amendment. The study was approved by the local bioethics committee (Bioethics Committee of the Medical University of Lublin, approval no KE-0254/229/2020). All participants were informed of the objectives of the study. They could have withdrawn at any stage of the examination. Each participant provided researchers with their written consent.

### 2.2. Study Protocol

#### 2.2.1. Assessment of Muscle Thickness

**Figure 1:**
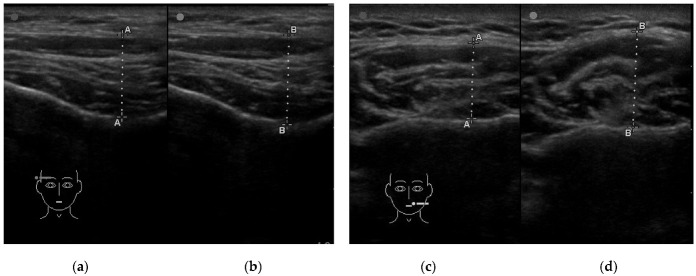
Example of an ultrasound examination of muscle thickness. (**a**) The temporalis muscle in the relaxed mandible position with slight contact between opposing teeth; (**b**) the temporalis muscle in the maximum voluntary clenching; (**c**) the masseter muscle in the relaxed mandible position with slight contact between opposing teeth; (**d**) the masseter muscle in the maximum voluntary clenching; line A-A—measuring line of the thickness of the muscle in the relaxed mandible position with slight contact between opposing teeth; line B-B—measuring line of the thickness of the maximum voluntary clenching.

The cross-sectional thickness of the muscles was assessed using an M-Turbo ultrasound machine equipped with a 15–6 MHz linear transducer with scan depth up to 6 cm (SonoSite Inc, Bothell, WA, USA). During the examination, patients were lying down on the dental chair in a horizontal position. The thickness of the masseter muscle was measured with an ultrasound probe positioned perpendicular to the mandibular ramus and in the occlusal plane [24]. The anterior part of the temporalis muscle was examined with a probe positioned in front of the hairline, parallel to and 1 cm above the zygomatic arch, perpendicular to the temporal bone [25]. The anterior belly of the digastric muscle was assessed with a probe positioned in the middle between the hyoid bone and the mental protuberance, perpendicular to the long axis of the muscle and the skin [26]. The thickness of the sternocleidomastoid muscle was examined with a probe positioned perpendicular to the long axis of the muscle and the skin, in the middle between the mastoid process and the clavicular notch of the sternum [27]. The cross-sectional thickness of all the examined muscles on both sides was measured in the relaxed mandible position with slight contact between opposing teeth (Rest) and during maximum voluntary clenching (Clench) (Figure 1). The whole procedure was performed twice for each patient. The thickness was measured in the thickest part of the muscle on the scan, directly on the scanner screen with 0.1 mm accuracy. The mean of the two measurements was included in the analysis. All tests were taken by the same examiner (author M.W.).

#### 2.2.2. Assessment of the Muscle Activity

Masticatory and neck muscle activity was performed using the 8-channel BioEMG III electromyograph compatible with the BioPAK measurement system (BioResearch Associates, Inc., Milwaukee, WI, USA). The subjects took a standardized position in the dental chair [16,28]. The study was conducted between 8 and 12 a.m. The skin of the subjects was cleaned with 90% ethanol, the surface of the electrodes placed on the skin (Ag/AgCl with a diameter of 30 mm and a conductive surface of 16 mm—SORIMEX, Torun, Poland) following the standards of the SENIAM program [29]. Placing electrodes was presented in the earlier study by the authors [16].

Four muscle pairs were analyzed: the anterior part of the temporalis muscle (TA), the superficial part of the masseter muscle (MM), the anterior belly of the digastric muscle (DA), and the middle part of the sternocleidomastoid muscle belly (SCM) [16,28]. Surface electrodes placement and electromyographic (sEMG) examination were taken by the same physiotherapist (author G.Z.).

**Figure 2:**
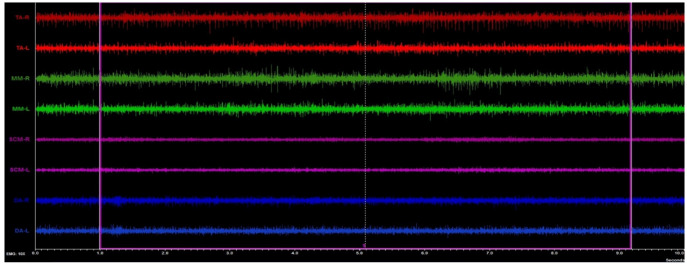
Example of the surface electromyography traces during resting activity.

The electromyography study was performed with the subjects’ eyes closed. This was dictated by the exclusion of the possible neurologic component [15,16,17]. The sEMG activity was recorded during resting mandibular position for 10 s. The sEMG signals obtained during the test were amplified and cleaned according to the previously described methodology [16]. The BioPAK Noise Tests were administered to all participants before and after each measurement. Automatic processing of the sEMG signal based on a root mean square calculation in BioPAK software produced an averaged measure of values, which then were used for muscle-activity analysis (Figure 2).

#### 2.2.3. Ophthalmic Examination

##### The Axial Length of the Eyeball

An IOL Master 500 (Carl Zeiss Meditec, Jena, Germany) was used to examine eyeball length. It obtains data based on the optical path distance from the anterior surface of the cornea to the retinal pigment epithelium. This device is used in clinical practice to calculate the power of artificial intraocular lenses. The IOL Master 500 is a noninvasive test and uses 780 nm partial coherence interferometry to measure the eye’s axial length [30]. All tests were taken by the same examiner (author A.M.-W.). All patients sat in front of the device head, resting their chin and forehead against the tripod. Patients were asked to perform a full blink just before the measurements were taken. This order was given to distribute an optically smooth tear film on the cornea. The eyes were focused when the instrument head was approximately 5.5 cm from the patient. Five separate measurements were taken and averaged for axial length [31].

##### The Thickness of the Retina and Choroid

**Figure 3:**
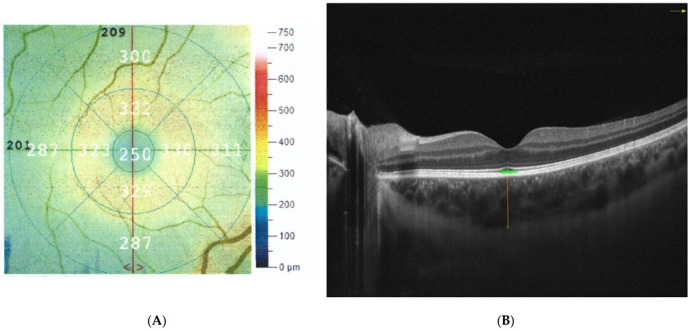
Example of a study of retinal thickness (**A**) and choroidal thickness (**B**).

Optical coherence tomography (OCT) was used to determine the thickness of the retina and choroid as well as to detect any possible changes in the macula. These measurements are used in a wide range of ophthalmic examinations to determine the thickness, volume, and structure of various retinal layers [32]. Chosen patients sat in front of the device head, resting their chin and forehead against the tripod. The study was performed with Optovue AngioVue (Fremont, CA, USA). To ensure the correctness, the cutoff point for scan quality was 7/10. The retinal and choroidal thickness were measured in the fovea. Area-identification and segmentation methods were automatically selected, and the thickness of the retina was measured in 6 × 6 mm scans. The central macular thickness (CMT) was characterized as the average thickness measured at the point of intersection of the six radial OCT scans. It was automatically measured by the OCT mapping system in the healthy eye. The central foveal thickness (CFT) was defined as the distance between the vitreoretinal interface and the anterior surface of the retinal pigment epithelium (RPE) in the center of fovea; this was measured manually and was also automated using the Optovue AngioVue measurement software. Mean retinal thickness was noted at the central 1 mm. This measurement was given by the automated software (Figure 3A) [33]. The choroidal thickness (CT) was measured manually with the inbuilt caliper in OCT cross-sectional scans. The CT measurement was performed perpendicular to the RPE, going from the posterior RPE edge to the choroid–scleral junction in the center of the fovea (Figure 3B) [33]. The retinal and choroidal thickness measurements were performed at the same time of day between 1 and 3 p.m. to eliminate the changes caused by the time of day. Evaluation of OCT scans and measurement of retinal and choroidal thickness were performed by two independent readers (A.M.-W. and M.R.) [32,34] (Figure 3).

##### The Intraocular Pressure

The final test was taken to determine the intraocular pressure. It was performed at the end of the whole examination so that the administered anesthesia and potential epithelial defects would not affect other measurements. The intraocular pressure was measured with a Tono-Pen XL (Medtronic Solan, FL, USA) in both eyes after the application of ALCAINE 0.5% (Alcon Laboratories Inc., Fort Worth, TX, USA). The apparatus was positioned vertically to the anesthetized cornea. The device uses voltage micrometer technology and a 1.0 mm transducer tip. The tip of the tonometer compresses the cornea, and its resistance depends on the pressure in the eyeball. The Tono-Pen XL gently shrinks the cornea and displays an average of four independent readings and a statistical factor [35,36].

#### 2.2.4. Statistical Analysis

For statistical analysis, IBM SPSS Statistics 13.3 program was used. First, the Shapiro–Wilk test and the Kolmogorov–Smirnov test (with Lillierfors correction) were applied to check the distribution. The Student’s *t*-test (t) or Mann–Whitney U test (z) was used to compare the differences between ophthalmological parameters, bioelectrical tension, and muscle thicknesses (TA, MM, SCM, and DA) in the study groups, depending on the distribution. The Spearman rho test (s) and r-Pearson test (p) (depending on the distribution) were used to analyze the correlation between the length of the eyeball, reticular thickness, choroidal thickness, intraocular pressure, and the thickness and bioelectrical tension of selected muscles of the masticatory system and the cervical segment. The Spearman rho test (s) and r-Pearson test varied between −1 (perfect negative monotonic association) and +1 (perfect positive monotonic association). A correlation was considered large for values greater than 0.5 and moderate for values between 0.3 and 0.5 [37]. Effect sizes were determined for the *t*-test using the Cohen d method and interpreted as small (0.2), medium (0.5), and large (0.8) effect sizes [38,39]. Statistical significance was set at *p* ≤ 0.05.

## 3. Results

The groups differed significantly in the axial length of the eyeball. The other parameters did not differ statistically (Table 1).

The results of the between-group analysis showed significant differences between TA Rest _R_ muscle thickness in clenching. A larger cross-sectional TA _R_ was observed in subjects with myopia. Moreover, a smaller SCM Rest _R_ and SCM Clench _R_ thickness was also observed in subjects with myopia in comparison to women without the refractive error (Table 2).

Correlation (right-hand) showed a negative correspondence between TA _R_ bioelectrical voltage and retinal thickness in the myopia group. In the group without refractive error, co-correlations were observed between ocular length and MM Rest _R_ and MM Clench _R_, retinal thickness and SCM Rest _R_ and SCM Clench, along with intraocular pressure and SCM _R_ bioelectrical activity (Table 3).

Correlation (left-hand) showed a correspondence between eyeball length and TA L bioelectric active, between choroidal thickness and TA L and SCM L bioelectric active, between intraocular pressure and DA Clench L in the myopia group. No left-hand correlations were observed in the no-refractive-error group (Table 4).

## 4. Discussion

This study is aimed to examine the correlations between masticatory and neck muscle thickness and activity versus eyeball length, retinal thickness, choroidal thickness, and intraocular pressure in healthy women versus women with myopia.

Subjects with myopia had lower SCM thickness compared to subjects without refractive error. In myopic subjects, single correlations were seen between eyeball length, retinal thickness, choroidal thickness, intraocular pressure, and masticatory muscle bioelectrical activity. There was a single correlation between muscle thickness during contraction and intraocular pressure, and it was on the border of a medium-impact effect. In the group without refractive error, correlations were observed with MM thickness versus ocular depth length and SCM thickness, retinal thickness, and intraocular pressure versus SCM bioelectrical activity.

The results obtained during the procedure confirm the interdependence between the stomatognathic system and the organ of vision. The analysis was performed with closed eyes in the test and control groups. This allowed the exclusion of the neurological component [15,16,17], and therefore, the results should be considered as functional/biomechanical or biochemical changes.

The standard for the axial length of the eyeball in healthy subjects is assumed to be about 23.75 mm [40,41]. The axial length of the eyeball is 24 mm for low myopia (−6 D < refractive error < 0 D), whereas the eyeball length for high myopia is approximately 30 mm (refractive error < −6 D) [42]. The study group included subjects with axial myopia, and their eyeball length was above 24 mm (Table 1). The retinal thickness of both eyes in the study group was greater than in the control group. According to a study by Zereid and Osuagwu, the retinal thickness measured in the fovea in healthy subjects is 238.5 ± 8.4, and in subjects with low myopia is 253.4 ± 8.4 at a mean age of 27 years [43]. Greater retinal thickness in subjects with myopia compared to those without refractive error was also recognized in the author’s study but did not reach the assumed level of significance. (Table 1). The observed thicknesses were also similar. In healthy subjects, the normal choroidal thickness is about 250 to 350 μm [44]. According to Hoseini-Yazdi’s studies, the thickness of the choroid changes with the refractive error; myopia subjects had a thinner choroid than people without refractive error [45]. This was seen unilaterally in the author’s study (the left choroid was thinner) compared to the treatment group. According to the research of Gupta et al., mean wake-up pressure was defined as 16.12 ± 2.94 for the group with myopia and 15.26 ± 2.74 for the group without refractive error [46]. Similar values were achieved in the author’s study of higher intraocular pressure in patients with myopia (Table 1). The above differences observed in the author’s study and the studies of other authors indicate significant changes in the subjects whose eyes did not have refractive error compared to the one with myopia. The main consideration should be whether the changes in the visual system are primary or secondary compared to the changes in the myofascial system. Changes in the axial length of the eyeball, retinal thickness, choroidal thickness, and intraocular pressure may indicate a mechanical component [17].

**Figure 4:**
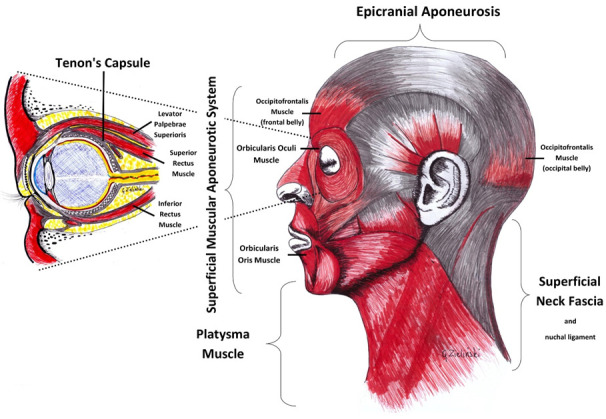
Scheme of anatomical compounds.

The connection between the visual organ and the muscles of the chewing organ can take place via a fascia network [17]. The Tenon’s fascia begins at the optic nerve sheath deep inside the orbit. It divides in this area into two parts: the first one extends anteriorly surrounding the eyeball up to the oculomotor attachment and further connects with the conjunctiva [47]. The second part forms on the meatus of the oculomotor muscles. It further connects to the orbital periosteum and the fascia of the eyelid elevator. This system of connections lets the eyeball see the appropriate shape [48]. The Tenon’s fascia surrounds six oculomotor muscles and connects with the fascia of the eyelid lever muscle [47,49]. It is not possible to separate the function of the eye muscle from the action of the Tenon fascia [47]. For three-dimensional vision to occur, the eye muscles must constantly be active and perform movements to adjust the visual organ to the objects to be observed [48]. The correct function of the optic organ and the axial length of the eyeball, the thickness of the retina, the structure of the choroid, and the intraocular pressure may depend strongly on the external and intraocular musculature. Muscle tension and its elasticity are related to fascia [17,48]. Tenon’s fascia surrounds six oculomotor muscles and connects with the fascia of the eyelid lever muscle [47,49]. The oculomotor belongs to the superficial musculoaponeurotic system (SMAS), including the facial muscle [50]. SMAS is combined with epicranial aponeurosis and successively with the cervical ligament. At the front, the SMAS connects with the superficial fascia surrounding the platysma muscle [47,50]. The temporal parietal fascia connects with the SMAS [51]. These connections can explain changes in the TA, MM, SCM, and DA muscles. (Figure 4).

The difference in TA thickness between the two groups may be associated with greater activation of these muscles in myopic subjects. What is also worth stressing is that uncorrected refractive errors very frequently coexist with headaches [52]. According to Marasini et al., refractive and binocular vision abnormalities should be investigated in detail in all patients with headaches [52]. One of the causes of headaches may be the activity of TA and increased tension in the epicranial aponeurosis [53,54]. The statistically significant differences between groups in SCM thickness may be related to greater SCM muscle activity in individuals with myopia. Patients often compensate for visual problems by leaning towards the object or turning their heads from side to side. Moreover, individuals with myopia often protract the head and cervical spine, leading to increased tension in the thoracic muscles, descending fibers of the quadriceps, scapular elevator, and sternocleidomastoid muscles [55]. The head position and mandibular movements (lateral flexion and tilt) have been perceived as the ones that affect SCM thickness and activity, especially in the clenched state [56]. Headaches associated with increased TA activity, epicranial aponeurosis, and a change in protracted alignment may be related to changes in proprioception. The cervical proprioceptive system (CPS) consists of mechanoreceptors located in the deep cervical fascia, ligaments, short cervical spine muscles, and intervertebral joints [57]. Information from the cervical region is combined with information received from the vestibular bite [58,59]. The activity of the vestibular bite in people with nearsightedness is specifically linked to the vestibulo-ocular reflex (VOR) [15,17]. People with myopia may experience changes in their perception of visual data. These inputs play an important role in the maintenance and modifications of muscle basal tone [60,61]. Afferent impulses from proprioceptors cooperate with labyrinthine impulses to support oculomotor muscle activity through the VOR [15]. The connection between the cervical region and changes in masticatory muscle activity has been confirmed [62]. It is worth considering whether the changes in the cervical area and tension of facial and cranial muscles are primary or secondary to the changes in the visual system. The influence of myopia on TA, MM, SCM, and DA activity is still not fully understood. An anatomical connection is undoubtedly discernible.

In subjects without refractive error, only unilateral correlations related to MM thickness compared to eyeball length and SCM versus retinal thickness were noted. The unilateral correlations may be related to a preference for the masticatory side [63,64]. Unilateral stimulation could also transmit tensions via the above-mentioned fascial pathway. The phenomenon needs further study.

The presented study has several limitations. Firstly, the group is homogeneous and should be expanded to include the male population. Secondly, the size of the examined group was small. Therefore, future studies should be prospectively examined on a larger age-diverse population. Moreover, refractive errors are also linked to race [65,66]. The analysis in this study involved Caucasian women. Prospective studies should be conducted on different races to compare their link. Thirdly, in the presented study, we used RDC/TMD criteria. Diagnostic criteria for TMDs were changed to The Diagnostic Criteria for Temporomandibular Disorders (DC/TMDs) in 2014. So far, DC/TMDs have not been translated into Polish.

## 5. Conclusions

Refractive errors are related to differences in masticatory and neck muscle thickness and activity. Bioelectrical activity within the temporalis anterior seems to be associated with ocular length, retinal thickness, and choroidal thickness in women with myopia.

## Figures and Tables

**Table 1:** Presentation of ophthalmological parameters between the groups.

		Myopia Subject (*n* = 21)		Subject without Refractive Error (*n* = 19)				
	Side	Mean	SD	Mean	SD	Test		*p*
Refractive Error _(Dsph)_	_R_	−3.00	0.50	NA	NA		NA	NA
	_L_	−3.00	1.50					
Axial Length _(mm)_	_R_	24.27	0.69	23.30	0.55	t	4.85	0.00 * ES = 1.56
	_L_	24.31	0.84	23.27	0.56	t	4.51	0.00 * ES = 1.46
Retinal Thickness _(μm)_	_R_	252.71	18.14	244.26	12.16	t	1.71	0.10
	_L_	245.95	31.10	242.11	11.89	z	1.30	0.19
Choroidal Thickness _(μm)_	_R_	349.05	82.86	343.32	82.53	t	0.22	0.83
	_L_	322.86	83.71	328.89	84.29	t	−0.23	0.82
Intraocular Pressure _(mmHg)_	_R_	16.10	4.07	15.11	4.29	t	0.75	0.46
	_L_	15.48	4.20	14.79	3.85	t	0.54	0.59

NA—not applicable; _Dsph_—diopters; _R_—right site; _L_—left site; _mm_—millimeters; _μm_—micrometers; _mmHg_—millimeter of mercury; t—student *t*-test; z—Mann–Whitney U test; ES—effect size; *—significant difference.

**Table 2:** Comparison of masticatory and cervical muscle thickness and activity between groups.

	Myopia Subject (*n* = 21)		Subject without Refractive Error (*n* = 19)		Test		*p*
	Mean	SD	Mean	SD			
TA Rest_R__(mm)_	13.18	1.78	12.20	1.48	t	1.90	0.06
TA Clench_R__(mm)_	13.86	1.82	12.77	1.49	t	2.06	0.04 * ES = 0.60
MM Rest_R__(mm)_	11.94	2.18	12.42	1.51	t	−0.81	0.42
MM Clench_R__(mm)_	13.48	2.39	13.52	1.58	t	−0.05	0.96
DA Rest_R__(mm)_	6.55	0.63	6.67	0.81	z	−0.33	0.75
DA Clench_R__(mm)_	6.47	0.55	6.67	0.83	z	−0.64	0.52
SCM Rest_R__(mm)_	9.36	1.37	10.25	1.37	t	−2.05	0.04 * ES = 0.65
SCM Clench_R__(mm)_	9.37	1.31	10.26	1.42	t	−2.06	0.04 * ES = 0.69
TA_R__(μV)_	1.80	0.84	2.05	1.99	z	0.66	0.51
MM_R__(μV)_	2.53	1.67	2.18	1.31	z	0.58	0.56
DA_R (μV)_	1.30	0.37	1.16	0.39	z	−0.96	0.34
SCM_R__(μV)_	1.70	0.75	1.93	0.76	z	1.57	0.12
TA Rest_L__(mm)_	12.25	1.78	12.20	1.90	z	−0.03	0.98
TA Clench_L__(mm)_	12.88	1.87	12.80	2.00	z	0.22	0.83
MM Rest_L__(mm)_	12.43	2.30	12.69	1.55	z	−1.02	0.31
MM Clench_L__(mm)_	13.78	2.40	13.75	1.53	z	−0.03	0.98
DA Rest_L__(mm)_	6.70	0.97	6.56	0.75	t	0.37	0.71
DA Clench_L__(mm)_	6.61	0.98	6.58	0.74	t	0.09	0.93
SCM Rest_L__(mm)_	9.51	1.55	10.00	1.14	t	−1.12	0.27
SCM Clench_L__(mm)_	9.56	1.55	9.99	1.15	t	−1.00	0.33
TA_L__(μV)_	1.90	0.93	2.30	1.80	z	−0.35	0.72
MM_L__(μV)_	2.01	1.09	2.40	1.65	z	−1.14	0.26
DA_L__(μV)_	1.40	0.49	1.33	0.45	z	−0.22	0.83
SCM_L__(μV)_	1.71	0.75	1.63	0.54	z	0.50	0.62

TA—the temporalis anterior; MM—the superficial part of the masseter muscle; SCM—the middle part of the sternocleidomastoid muscle; DA—the anterior belly of the digastric muscle; _R_—right site; _L_—left site; _μV_—microvolt; _mm_—millimeters; Rest—ultrasound muscles examination in the relaxed mandible position with slight contact between opposing teeth; Clench—ultrasound muscles examination in the maximum voluntary clenching; t—student *t*-test; z—Mann–Whitney U test; ES—effect size; *—significant difference.

**Table 3:** Presentation of the resulting right-hand correlations between groups.

		Myopia Subject		Subject without Refractive Error	
		r	*p*	r	*p*
Axial Length_R_ _(mm)_	TA Rest_R (mm)_	0.18 ^p^	0.43	0.33 ^p^	0.17
	TA Clench_R (mm)_	0.24 ^p^	0.30	0.32 ^p^	0.19
	MM Rest_R (mm)_	0.08 ^p^	0.72	0.52 ^p^	0.02 *
	MM Clench_R (mm)_	0.06 ^p^	0.80	0.62 ^p^	0.00 *
	DA Rest_R (mm)_	0.28 ^s^	0.21	−0.05 ^p^	0.85
	DA Clench_R (mm)_	0.18 ^s^	0.43	−0.03 ^p^	0.92
	SCM Rest_R (mm)_	−0.23 ^p^	0.31	−0.28 ^p^	0.24
	SCM Clench_R (mm)_	−0.23 ^p^	0.32	−0.30 ^p^	0.21
	TA_R (μV)_	−0.24 ^s^	0.29	0.14 ^s^	0.57
	MM_R (μV)_	0.34 ^s^	0.13	−0.03 ^s^	0.90
	DA_R (μV)_	−0.23 ^p^	0.32	0.20 ^s^	0.41
	SCM_R (μV)_	0.29 ^p^	0.20	0.23 ^s^	0.35
Retinal Thickness _R_ _(__μm)_	TA Rest_R (mm)_	0.40 ^p^	0.07	−0.15 ^p^	0.53
	TA Clench_R (mm)_	0.38 ^p^	0.09	−0.24 ^p^	0.32
	MM Rest_R (mm)_	−0.28 ^p^	0.21	−0.17 ^p^	0.49
	MM Clench_R (mm)_	−0.09 ^p^	0.69	−0.06 ^p^	0.82
	DA Rest_R (mm)_	−0.09 ^s^	0.71	−0.03 ^p^	0.89
	DA Clench_R (mm)_	−0.13 ^s^	0.59	−0.07 ^p^	0.76
	SCM Rest_R (mm)_	−0.26 ^p^	0.25	−0.47 ^p^	0.04 *
	SCM Clench_R (mm)_	−0.25 ^p^	0.27	−0.48 ^p^	0.04 *
	TA_R (μV)_	−0.52 ^s^	0.02 *	−0.21 ^s^	0.39
	MM_R (μV)_	−0.23 ^s^	0.31	0.12 ^s^	0.62
	DA_R (μV)_	−0.28 ^p^	0.22	0.36 ^s^	0.13
	SCM_R (μV)_	−0.03 ^p^	0.91	0.07 ^s^	0.77
Choroidal Thickness _R_ _(__μm)_	TA Rest_R (mm)_	0.38 ^p^	0.09	−0.17 ^p^	0.48
	TA Clench_R (mm)_	0.37 ^p^	0.10	−0.16 ^p^	0.51
	MM Rest_R (mm)_	−0.08 ^p^	0.72	−0.33 ^p^	0.17
	MM Clench_R (mm)_	0.02 ^p^	0.92	−0.22 ^p^	0.36
	DA Rest_R (mm)_	−0.19 ^s^	0.41	−0.13 ^p^	0.59
	DA Clench_R (mm)_	−0.28 ^s^	0.23	−0.14 ^p^	0.56
	SCM Rest_R (mm)_	−0.09 ^p^	0.69	0.18 ^p^	0.46
	SCM Clench_R (mm)_	−0.13 ^p^	0.57	0.19 ^p^	0.44
	TA_R (μV)_	0.29 ^s^	0.21	−0.28 ^s^	0.24
	MM_R (μV)_	0.19 ^s^	0.41	−0.10 ^s^	0.69
	DA_R (μV)_	0.10 ^p^	0.66	0.08 ^s^	0.74
	SCM_R (μV)_	0.37 ^p^	0.09	−0.21 ^s^	0.39
Intraocular Pressure_R_ _(mmHg)_	TA Rest_R (mm)_	0.16 ^p^	0.49	0.37 ^p^	0.11
	TA Clench_R (mm)_	0.18 ^p^	0.43	0.34 ^p^	0.15
	MM Rest_R (mm)_	−0.04 ^p^	0.88	−0.07 ^p^	0.78
	MM Clench_R (mm)_	0.09 ^p^	0.70	−0.08 ^p^	0.74
	DA Rest_R (mm)_	0.36 ^s^	0.11	−0.14 ^p^	0.58
	DA Clench_R (mm)_	0.40 ^s^	0.08	−0.10 ^p^	0.69
	SCM Rest_R (mm)_	−0.23 ^p^	0.31	−0.14 ^p^	0.57
	SCM Clench_R (mm)_	−0.23 ^p^	0.31	−0.15 ^p^	0.55
	TA_R (μV)_	0.33 ^s^	0.14	0.10 ^s^	0.68
	MM_R (μV)_	0.08 ^s^	0.74	−0.31 ^s^	0.19
	DA_R (μV)_	0.17 ^p^	0.46	0.07 ^s^	0.78
	SCM_R (μV)_	−0.20 ^p^	0.38	−0.49 ^s^	0.03 *

TA—the temporalis anterior; MM—the superficial part of the masseter muscle; SCM—the middle part of the sternocleidomastoid muscle; DA—the anterior belly of the digastric muscle; _R_—right site; _μV_—microvolt; _mm_—millimeters; _μm_—micrometers; _mmHg_—millimeter of mercury; Rest—ultrasound muscles examination in the relaxed mandible position with slight contact between opposing teeth; Clench—ultrasound muscles examination in the maximum voluntary clenching; ^s^—the Spearman rho test; ^p^—the Pearson test; ES—effect size; *—significant difference.

**Table 4:** Presentation of the resulting left-hand correlations between groups.

		Myopia Subject		Subject without Refractive Error	
		r	*p*	r	*p*
Axial Length_L_ _(mm)_	TA Rest_L (mm)_	−0.08 ^p^	0.72	0.28 ^s^	0.25
	TA Clench_L (mm)_	0.18 ^p^	0.43	0.32 ^s^	0.19
	MM Rest_L (mm)_	0.27 ^s^	0.23	0.21 ^p^	0.39
	MM Clench_L (mm)_	0.19 ^p^	0.41	0.38 ^p^	0.10
	DA Rest_L (mm)_	0.12 ^p^	0.60	−0.22 ^s^	0.37
	DA Clench_L (mm)_	0.06 ^p^	0.79	−0.30 ^p^	0.21
	SCM Rest_L (mm)_	−0.23 ^p^	0.33	0.03 ^p^	0.91
	SCM Clench_L (mm)_	−0.13 ^s^	0.58	0.02 ^p^	0.94
	TA_L (μV)_	−0.76 ^s^	0.00 *	0.00 ^s^	1.00
	MM_L (μV)_	0.03 ^s^	0.91	0.21 ^s^	0.38
	DA_L (μV)_	−0.12 ^s^	0.61	0.43 ^p^	0.07
	SCM_L (μV)_	−0.37 ^s^	0.10	0.36 ^s^	0.13
Retinal Thickness_L_ _(μm)_	TA Rest_L (mm)_	−0.16 ^s^	0.48	−0.06 ^s^	0.80
	TA Clench_L (mm)_	−0.09 ^s^	0.68	−0.03 ^s^	0.92
	MM Rest_L (mm)_	0.21 ^s^	0.37	0.00 ^p^	1.00
	MM Clench_L (mm)_	0.10 ^s^	0.68	0.03 ^p^	0.89
	DA Rest_L (mm)_	0.07 ^s^	0.76	0.04 ^s^	0.86
	DA Clench_L (mm)_	−0.05 ^s^	0.83	0.01 ^p^	0.98
	SCM Rest_L (mm)_	−0.15 ^s^	0.53	−0.10 ^p^	0.68
	SCM Clench_L (mm)_	−0.12 ^s^	0.59	−0.11 ^p^	0.65
	TA_L (μV)_	−0.26 ^s^	0.25	−0.11 ^s^	0.66
	MM_L (μV)_	−0.17 ^s^	0.45	−0.05 ^s^	0.85
	DA_L (μV)_	0.07 ^s^	0.77	0.39 ^p^	0.09
	SCM_L (μV)_	−0.12 ^s^	0.60	0.00 ^s^	0.99
Choroidal thickness_L_ _(μm)_	TA Rest_L (mm)_	0.06 ^p^	0.80	0.17 ^s^	0.48
	TA Clench_L (mm)_	−0.15 ^p^	0.52	0.09 ^s^	0.73
	MM Rest_L (mm)_	−0.19 ^s^	0.40	−0.31 ^p^	0.20
	MM Clench_L (mm)_	−0.21 ^p^	0.36	−0.20 ^p^	0.41
	DA Rest_L (mm)_	−0.18 ^p^	0.42	−0.16 ^s^	0.50
	DA Clench_L (mm)_	0.04 ^p^	0.87	−0.22 ^p^	0.36
	SCM Rest_L (mm)_	−0.01 ^p^	0.97	0.28 ^p^	0.24
	SCM Clench_L (mm)_	−0.04 ^s^	0.88	0.29 ^p^	0.23
	TA_L (μV)_	0.53 ^s^	0.01 *	−0.27 ^s^	0.26
	MM_L (μV)_	0.09 ^s^	0.71	0.08 ^s^	0.75
	DA_L (μV)_	0.06 ^s^	0.78	0.12	0.63
	SCM_L (μV)_	0.46 ^s^	0.04 *	0.20 ^s^	0.42
Intraocular Pressure _L_ _(mmHg)_	TA Rest_L (mm)_	0.35 ^p^	0.12	0.09 ^s^	0.71
	TA Clench_L (mm)_	0.29 ^p^	0.20	0.09 ^s^	0.71
	MM Rest_L (mm)_	−0.10 ^s^	0.66	0.11 ^p^	0.65
	MM Clench_L (mm)_	0.08 ^p^	0.73	0.13 ^p^	0.58
	DA Rest_L (mm)_	0.43 ^p^	0.05	0.39 ^s^	0.10
	DA Clench_L (mm)_	0.48 ^p^	0.03 *	0.36 ^p^	0.14
	SCM Rest_L (mm)_	−0.13 ^p^	0.58	0.08 ^p^	0.75
	SCM Clench_L (mm)_	−0.24 ^s^	0.30	0.07 ^p^	0.77
	TA_L (μV)_	0.22 ^s^	0.33	−0.11 ^s^	0.65
	MM_L (μV)_	−0.04 ^s^	0.88	−0.08 ^s^	0.73
	DA_L (μV)_	−0.31 ^s^	0.17	0.06 ^p^	0.79
	SCM_L (μV)_	0.16 ^s^	0.50	−0.17 ^s^	0.48

TA—the temporalis anterior; MM—the superficial part of the masseter muscle; SCM—the middle part of the sternocleidomastoid muscle; DA—the anterior belly of the digastric muscle; _L_—left site; _μV_—microvolt; _mm_—millimeters; _μm_—micrometers, _mmHg_—millimeter of mercury; Rest—ultrasound muscles examination in the relaxed mandible position with slight contact between opposing teeth; Clench—ultrasound muscles examination in the maximum voluntary clenching; ^s^—the Spearman rho test; ^p^—the Pearson test; ES—effect size; *—significant difference.

## Data Availability

The datasets generated during and/or analyzed during the current study are available from the corresponding author on reasonable request.

## Abbreviations

_μm_	micrometers
_μV_	microvolt
CFT	central foveal thickness
Clench	ultrasound muscles examination in the maximum voluntary clenching
CPS	cervical proprioceptive system
DA	anterior belly of the digastric muscle
_Dsph_	Diopters
IOP	intraocular pressure
_L_	Left site
_mm_	millimeters
MM	the superficial part of the masseter muscle
_mmHg_	millimeter of mercury
^p^	Pearson test
_R_	Right site
RDC/TMD	Research Diagnostic Criteria for Temporomandibular Disorders
Rest	ultrasound muscles examination in the relaxed mandible position with slight contact between opposing teeth
^s^	Spearman rho test
SCM	middle part of the sternocleidomastoid muscle belly
sEMG	surface electromyography
SMAS	superficial musculoaponeurotic system
TA	part of the temporalis muscle
TMD	temporomandibular disorders
VOR	vestibulo-ocular reflex
